# Effect of morphology on the biomechanics of contusion models of non-human primate spinal cord injury: a finite element study in a digital population

**DOI:** 10.1371/journal.pone.0337794

**Published:** 2026-02-04

**Authors:** Numaira Obaid, Dexter L. Zamora, Cesar Jimenez-Gonzalez, Carolyn J. Sparrey

**Affiliations:** 1 School of Mechatronic Systems Engineering, Simon Fraser University, Surrey, British Columbia, Canada; 2 International Collaboration on Repair Discoveries (ICORD), Vancouver, Canada; University of Florida, UNITED STATES OF AMERICA

## Abstract

Traumatic spinal cord injuries (SCIs) stem from mechanical events that translate external forces through the spinal column, damaging the spinal cord. Since tissue damage is related to the strain/stress it experiences, finite element models are being increasingly used to supplement pre-clinical models of animal SCI. Simulations; however, are often conducted in a single geometry, while morphological variability has been highlighted as having an important influence on biomechanical outcomes. We developed tissue scale finite element models of non-human primate spinal cord injury with different morphologies (N = 40) to assess the effect of morphology on biomechanical outcomes. Applying the same displacement to different digital subjects generated different peak forces, and the magnitude of these forces was related to subject morphology, specifically the area of cerebrospinal fluid (CSF) and the occlusion of the spinal canal by the spinal cord (SCO/SC), particularly in the mediolateral direction (SCOW/SCW). Despite the same loading (0.75 N preload and 4-mm displacement at 500 mm/s), different subjects experienced a wide range of impact forces (13–33 N) due to morphological differences. In pre-clinical experiments, this variability could lead to drastically different outcomes, ranging from no functional deficits at the lower end (13 N) to unintended contralateral contusions at the higher end (33 N), despite the intent to induce unilateral injury. Peak forces were statistically correlated with white matter sparing, which affects observed functional outcomes. We showed that both tissue-level and impact biomechanics are significantly affected by morphology, emphasizing the need to include diversity and morphological variability into computational models of spinal cord injury. This highlights that either impact parameters need to be adjusted for morphological variability or that animals should be pre-screened for cord/column morphology, which can be prohibitively expensive. Future work is needed to determine how to scale these impact parameters for different morphologies.

## Introduction

Spinal cord injuries (SCIs) stem from a mechanical event, where an external force translates through the spinal column damaging the constituent tissues of the spinal cord [1–3]. Since the tissue-level biomechanics of human SCI are complex and rarely known, animal contusion injury models are used to replicate the injury mechanism in a pre-clinical setting. Pre-clinical testing of therapeutics requires the creation of reliable functional deficits between animal subjects, to improve the ability to detect beneficial therapeutic effects consistently and reproducibly. However, even in carefully designed animal experiments, sources of variability are common, and prior studies have reported variability in both functional outcomes and tissue damage even under the same impact settings [4–6]. Variability in pre-clinical experiments is typically decreased by carefully controlling the subject population, such as species strain, weight, and age to ensure that morphological differences are limited [7]. Due to the higher morphological variability in larger animals [8], particularly in the spinal canal [9], this approach is unfeasible in large animal models such as non-human primates (NHP). Additionally, in large animals such as NHPs, ethical and financial constraints limit the number of subjects used in a study. Thus, additional studies are required to investigate and reduce inherent sources of variability. However, experiments that parametrically examine the effect of different factors on variability are prohibitive to conduct systematically in large animals motivating the need for computational model systems.

SCIs occur due to mechanical tissue loading; therefore, prior studies have used finite element models to establish correlations between tissue loading (stress and strain) and histologically observed damage [1,10–12]. Parametric studies using finite element methods enable the systematic exploration of the effects of different experimental and subject variables on spinal cord injury biomechanics. Sparrey et al. (2016) used finite element models to show that submillimeter changes in mediolateral alignment could increase the impact forces in a unilateral cervical SCI contusion model by over 50% [2]. Fournely et al. (2020) examined the effect of spinal cord curvature, impactor position and inclination, and material properties in a finite element model of a murine cervical contusion injury [13] and found that material properties were the most significant influencer of tissue-level stresses and strains. Zhu et al. (2020) examined the effect of compression depth [14]. Frantsuzov et al. (2023) examined the effect of impactor velocity, depth, and geometry on the tissue stresses/strains generated in the spinal cord in a rat contusion finite element model of a thoracic spinal cord injury, emphasizing the role of impactor geometry [15]. In our prior study, we examined the effect of impactor diameter and mediolateral alignment on peak forces of ten NHP subjects and highlighted the importance of using subject-specific impact protocols [16].

While computational models have informed our understanding of different influencing parameters [13,17–21], one of the key limitations is that these studies are often conducted in a single generic morphology [13,16,22]. Even in studies that have explored the role of morphology, these are limited to a few discrete values. For example, Fournely et al. (2020) explored the effect of three spinal cord diameters [13]. While the study provided important insights into the effect of morphology, morphological variability is multidimensional and other factors such as the roundness of the cord and its fit within the spinal canal were not examined. Prior pre-clinical studies in rodent and porcine models have emphasized the role of intersubject morphology as a key factor influencing spinal cord injury outcomes [4]. In different parts of the body that have been studied using computational models, there is an emerging interest to integrate morphological diversity into these models to make findings more translatable [23,24]. However, similar studies have not been conducted to integrate morphological variability into computational models of spinal cord injuries. Investigating how morphology influences biomechanical outcomes could be essential in informing whether impact protocols should be modified to improve consistency, and how they should be modified. This knowledge could be essential in reducing experimental variability and producing consistent functional deficits for the testing of therapeutics.

In this study, our objective is to explore the effect of morphological variability on the tissue-level biomechanics in a NHP spinal cord injury model. By leveraging the relationship between tissue loading and damage, we will use computational models to investigate how morphological differences across subjects influences tissue loading patterns. This approach allows us to explore the influence of morphology, which is normally not feasible using pre-clinical studies due to practical and ethical limitations of experiments in NHPs.

## Methods

In this study, we employ a novel approach that utilizes a large digital NHP population with varying spinal column and canal morphology to investigate how morphological variability influences impact and tissue-level biomechanics in a unilateral contusion model of a non-human primate spinal cord injury

### Geometry

A method to generate a digital NHP geometry has been developed and reported previously [16,22] but was not used to investigate tissue-level biomechanics in different digital subjects under the same loading. In summary, pre-injury magnetic resonance imaging (MRI) was used to quantify the average and range of values for various metrics including the anterior-posterior and mediolateral diameters of the spinal cord and canal (SCOD, SCOW, SCD, and SCW). A Python script was used to generate geometries within these values, such that each time the script was run, values for each parameter were randomly selected and a new and different geometry was created. These values altered the shape of the overall spinal cord, primarily in the white matter, while the gray matter geometry remained the same. The code was run several times to generate a digital population set (N = 40) for computational analysis and the corresponding morphology for each digital subject was recorded (S1 Table). The population subset used in this study had an average mediolateral spinal canal (SCW) and spinal cord diameter (SCOW) of 11.6 ± 0.9 mm and 9.8 ± 0.7 mm, respectively. The average anterior-posterior spinal canal (SCD) and spinal cord diameter (SCOD) was 8.1 ± 0.4 mm and 6.3 ± 0.4 mm, respectively. These values are within the range of diameters reported for primates previously reported for the same primate species [9].

For each subject, the parameters (SCOD, SCOW, SCD, and SCW) were combined to calculate additional morphological parameters for statistical analysis including SCO and SC, the areas of the spinal cord and spinal canal based on the equation of the area of an ellipse with major and minor axes as SCOW and SCOD, respectively. The CSF Area was calculated as the difference between the two areas (SC – SCO). Similarly, the mediolateral and anterior-posterior CSF (CSF ML and CSF AP) were calculated as the difference between the spinal canal and spinal cord diameters, respectively.

### Computational model

**Fig 1:**
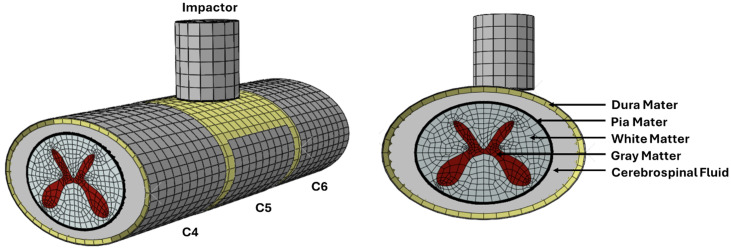
The computational model used in this study consisted of the spinal cord, partitioned into its constituent tissues, the dura and pia maters, the vertebral bodies and the cerebrospinal fluid.

A unilateral contusion impact was simulated based on our experimental protocol described in prior studies [5], see Fig 1. Briefly, the experimental protocol involves two steps: (1) a slow displacement of the spinal cord until it is compressed against the spinal canal, allowing cerebrospinal fluid to shift rostrally and caudally; followed by (2) a rapid displacement of the impactor to induce the contusion. The injury was simulated using FE analysis (Abaqus/CAE 2021, Simulia, Dassault Systemes Inc., Vancouver, Canada) based on our previously published computational model that has been validated against our NHP experiments [16,22,25]. The C4-C6 segment of the cervical spinal canal was modeled as discrete rigid bodies, where the C5 was modified to represent a partial laminectomy offset from the midline to expose the lateral side of the cord. The spinal cord was modeled as a three-dimensional solid component, partitioned into the grey and white matters, with the pia mater modeled as a 150-µm skin extending from the outer surface of the spinal cord [26]. The dura mater was defined as a 350 µm thick shell [27]. An offset was defined into the geometries to ensure that overlap and contact overclosure did not result from the thicknesses of each shell/skin. The space between the dura and pia maters was defined as the cerebrospinal fluid, represented using smoothed particle hydrodynamics (SPH). A 5-mm diameter beveled cylindrical discrete rigid impactor delivered a contusion at the C5 level, positioned 0.5-mm over the spinal cord midline.

A mesh refinement analysis was conducted to determine the appropriate element size assigned to the model. The spinal cord was a three-dimensional solid and was assigned C3D8R elements for the white and gray matter, and S4R elements for the pia mater, the cerebrospinal fluid was assigned C3D8R elements that converted into SPH particles instantaneously at the start of the simulation, and S4R elements were assigned to the dura mater. All material models, boundary conditions, and contact interactions have been previously validated and are described in greater detail in our prior studies [10,16,22,25,28] (Table 1).

**Table 1:** Material models used in this study.

	Density (kg/m^3^)	Elastic Region	Viscoelastic Region	
**White Matter** [10]	1041	Mooney-Rivlin QLV $C_{\text{1}\text{0}}=\text{3}\text{2}\text{7}\text{0}\;kPa$ $C_{\text{0}\text{1}}=\text{9}\text{1}\text{0}\;kPa$ D=239.3 kPa γ=6172 kPa v=0.49	$g_{\text{1}}=\text{0}.\text{5}\text{2}\text{5}\text{6}$ $g_{\text{2}}=\text{0}.\text{3}\text{1}\text{6}\text{3}$ $g_{\text{3}}=\text{0}.\text{1}\text{2}\text{5}$ $g_{\text{4}}=\text{0}.\text{0}\text{0}\text{7}$	$\tau_{\text{1}}=\text{0}.\text{0}\text{1}$ $\tau_{\text{2}}=\text{0}.\text{0}\text{2}$ $\tau_{\text{3}}=\text{0}.\text{2}$ $\tau_{\text{4}}=\text{2}.\text{0}$
**Gray Matter** [10]	1045	Hyperelastic, Ogden $\mu_{\text{1}}=\text{3}\text{4}.\text{7}\;kPa$ ∝=7.61 v=0.45	$g_{\text{1}}=\text{0}.\text{4}\text{1}\text{8}\text{3}$ $g_{\text{2}}=\text{0}.\text{2}\text{2}\text{5}\text{2}$ $g_{\text{3}}=\text{0}.\text{1}\text{2}\text{1}\text{3}$	$\tau_{\text{1}}=\text{0}.\text{6}\text{4}$ $\tau_{\text{2}}=\text{6}.\text{4}$ $\tau_{\text{3}}=\text{6}\text{4}.\text{0}$
**Pia Mater** [29]	1075	E=39.3 MPa v=0.3	–	
**Dura Mater** [30]	1174	Hyperelastic, Ogden $G_{\text{0}}=\text{3}\text{2}\text{5}\text{0}\;kPa$ ∝=16.2 v=0.45	$g_{\text{1}}=\text{0}.\text{3}\text{1}\text{8}$ $g_{\text{2}}=\text{0}.\text{1}\text{2}\text{8}$ $g_{\text{3}}=\text{0}.\text{0}\text{9}\text{9}\text{7}$ $g_{\text{4}}=\text{0}.\text{0}\text{9}\text{9}\text{7}$	$\tau_{\text{1}}=\text{0}.\text{0}\text{0}\text{0}\text{9}$ $\tau_{\text{2}}=\text{0}.\text{0}\text{8}\text{1}$ $\tau_{\text{3}}=\text{0}.\text{5}\text{6}\text{4}$ $\tau_{\text{4}}=\text{4}.\text{6}\text{9}$
**Cerebrospinal Fluid** [31,32]	1007	c0=1381.7 s=1.979 $\Gamma_{\text{0}}=\text{0}.\text{1}\text{1}$	–	

The contusion impact was simulated to mimic the NHP unilateral cervical contusion experiments via two dynamic, explicit steps: (1) a preload phase, where the CSF was slowly displaced (30 mm/s) by the impactor until the cord was entrapped against the spinal canal and the preload force reached 0.75 N, and (2) an impact phase, where a high-speed contusion (approximately 500 mm/s to 4-mm displacement) was delivered. During the preload phase, only the rostral-caudal movement of cord and dura was restricted. The ends of the cord and dura were fully constrained during the impact phase. The CSF was allowed to flow in the rostral-caudal direction during the preload phase but not during the impact phase, representing CSF pressurization during a high-speed impact, as per our prior studies [10,33]. Peak impact forces were extracted as the primary outcomes.

### Tissue sparing

Tissue sparing was calculated individually for the white and gray matters of the cord at the epicenter as the percent volume of each tissue with a minimum principal logarithmic strain (min LEP) that exceeded previously defined injury thresholds. The transverse epicenter was defined as the location of the spinal cord immediately below the center of the impactor to ensure consistency between subjects. Different injury thresholds were used for each tissue based on our prior studies [1,10], which correlated minimum logarithmic strains with histological patterns of tissue damage in NHP [1,10]. In these studies, a strain level lower than *−0.44* in the gray matter and lower than *−0.77* in the white matter represented at least a *50%* probability of the tissue being damaged [33]; these values were selected from a range of tissue damage thresholds reported in our prior work [10,33]. At the epicenter, the volume of each element (EVOL) and its minimum principal logarithmic strain was extracted from each element for each material to calculate tissue sparing. Any elements in the gray or white matter with a strain value that exceeded the threshold was defined as injured. The percent of predicted damaged tissue was defined as a ratio of the sum of the injured element volumes to the total volume of that tissue. The percentage of spared tissue was defined as the remainder; these values were segregated for each tissue (gray and white matter) at the epicenter.

## Results

### Mesh convergence

A mesh convergence study was conducted on the entire model to select the appropriate mesh density used in the model. Four different finite element models were created with different element densities, and its effect on the value of the peak force (the maximum magnitude of force during the impactor displacement) was examined. The difference between the value predicted by the finest mesh (113,440 elements) and the second finest mesh (30,476 elements) was less than 1%, while the computational time increased by over six times. Thus, the second finest mesh was used in this study to optimize accuracy and computational efficiency. Tissue-level mesh verification was conducted by comparing the minimum principal logarithmic strain values of the white and gray matters, and a difference between the selected mesh and the finest mesh was between 2–4%, thus, the mesh was deemed sufficiently accurate.

### Population biomechanics

**Fig 2:**
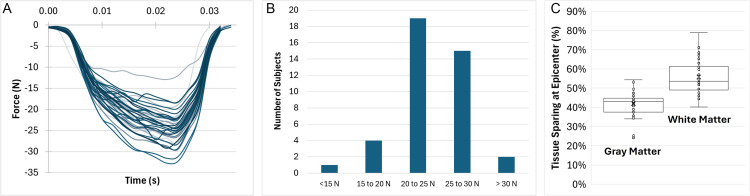
Variability in biomechanics and tissue sparing caused by morphology. Incorporating morphological variability into this computational model showed that the model is sensitive to changes in geometry, resulting in variability in the biomechanical outcomes including the forces (A), peak force (B), and tissue sparing (C).

We observed a normally distributed peak force based on this digital population (N = 40, indicated by S with the accompanying sample number), with an average peak force of 24.3 N ± 3.8 N, ranging from 12.9 N to 32.7 N (Fig 2A–B). The shape of the force-time curve over the duration of the impact remained relatively similar between subjects. Applying the same impact protocol (5-mm impactor at a 0.5-mm mediolateral alignment) to each subject produced a 15% variability in the peak force experienced by the NHP spinal cord during the contusion. Peak force values were binned in 5-N increments, and it was observed to be normally distributed (Fig 2B) with a few subjects resulting in substantially higher (S28 and S32) or lower (S14) peak forces than average values. In all subjects, the gray matter of the spinal cord experienced more predicted damage than the white matter, with an average epicenter tissue sparing (percent of predicted undamaged tissue area compared will total tissue cross sectional area) of 42% and 56% for the gray and white matters, respectively (Fig 2C). Predicted white matter tissue damage was more variable between the subjects than the gray matter damage (coefficient of variation of 9% vs 6%). The highest and lowest white matter tissue sparing were predicted for subject S14 (80%) and subject S20 (40%), respectively, while the highest and lowest tissue sparing in the gray matter was predicted for subject S28 (54%) and S20 (25%), respectively.

### Effect of morphology on peak force and impulse

**Fig 3:**
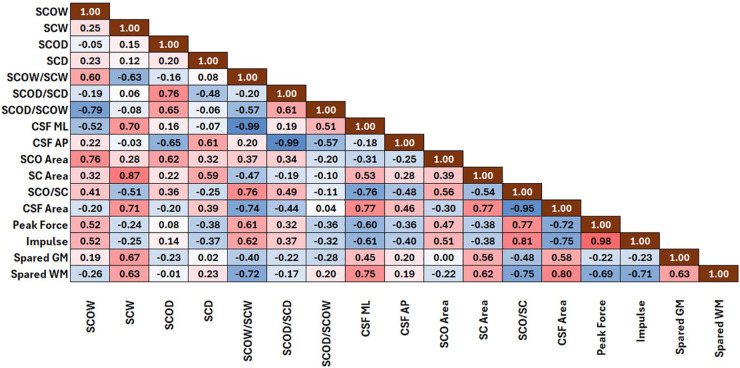
Correlation matrix between morphology and biomechanical measures. A heat map of the correlation matrix showing the linear relationship between the morphological and biomechanical measures. Pearson correlation coefficients are shown, with positive correlations shown in red and negative correlations shown in blue. A higher correlation coefficient is indicated by darker shades in both directions (red and blue). Morphology measures include the spinal cord diameters (SCOW and SCOD), spinal canal diameters (SCW and SCD), the thickness of the cerebrospinal fluid in the mediolateral (CSF ML) and anterior-posterior axes (CSF AP), and the transverse area of the spinal cord (SCO) and spinal canal (SC).

Peak forces resulting from the impact were most strongly correlated (*r = 0.77*) with the occlusion of the spinal canal by the spinal cord (SCO/SC), indicating that when the cross-sectional space available to the spinal cord was reduced, the forces that it experienced during an impact were higher (Fig 3). Greater canal occlusion was also related to reduced cerebrospinal fluid area (*r = −0.72*), which was strongly correlated with peak force, emphasizing the mechanical protection offered by the presence of CSF during impact.

**Fig 4:**
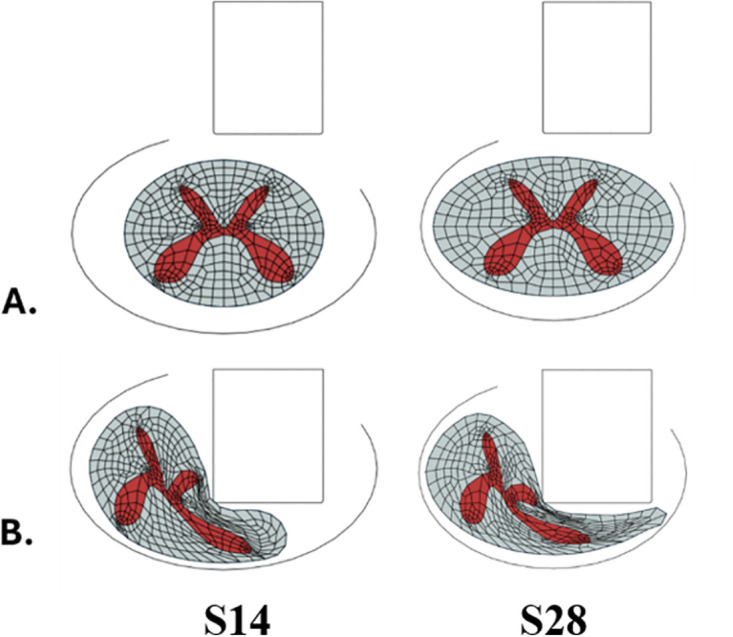
Impact of mediolateral geometry on spinal cord-impactor interaction. The mediolateral geometry of the spinal cord influences alters how it engages with the impactor. Increased CSF surrounding the spinal cord allowed the cord to move laterally during impact and reduced the forces applied to the spinal cord (S14), while a high spinal occlusion resulted in more tissue being engaged under the impactor and a higher peak force (S28).

The mediolateral occlusion of the canal (SCOW/SCW) was more significant than the anterior-posterior occlusion (SCOD/SCD) and was statistically correlated with the peak forces (*r = 0.61*), impulse (*r = 0.62)*, and white matter sparing (*r = −0.72*). We further observed that S14, the subject that experienced the lowest peak forces during impact, had one of the lowest SCOW/SCW ratios in the subset, while S28 and S32, the subjects with the highest peak forces, had the highest SCOW/SCW ratios. Other subjects with low SCOW/SCW ratios (S31 and S42) also had peak forces below 20 N. Fig 4 compares the deformed shape of the spinal cord of S14 and S28 during impact.

### Effect of morphology on tissue mechanics and predicted damage

At the injury epicenter, the predicted percentage of spared gray matter was positively correlated with the area of the cerebrospinal fluid (*r = 0.58*) (Fig 3). The predicted spared gray matter was also correlated with the spinal canal diameter in the mediolateral direction (*r = 0.67*) and the overall canal area (*r = 0.56)*, indicating that a larger canal predicted higher gray matter sparing. However, there was an inverse relationship with the fit of the cord within the canal, demonstrating that more occlusion of the canal by the spinal cord decreased the amount of gray matter spared. These correlations with morphology, however, were not as strong as that of the spared white matter. White matter sparing was highly correlated with morphology, demonstrating a strong relationship with mediolateral occlusion (SCOW/SCW, *r = −0.72*), mediolateral CSF thickness (*r = 0.75*), and the area of the CSF (*r = 0.80*). Once again, the anterior-posterior cord morphology presented a weaker statistical correlation with the gray or white matter sparing (*r = −0.23 and −0.01)*. Peak force and impulse were also important predictors of the white matter sparing, indicating that higher forces and impulse predicted higher tissue damage (*r = −0.69* and *r = −0.71*); however, gray matter sparing was not significantly correlated with these parameters. One possible explanation is that morphological changes were primarily reflected in the white matter in this study, while the gray matter morphology remained unchanged. The unilateral nature of the impact may have caused the gray matter to shift laterally, resulting in less direct interaction with the impactor compared to the surrounding white matter. Future studies should explore the relationship between gray mater sparing and biomechanics.

### Predicted tissue damage on the ipsilateral side

**Fig 5:**
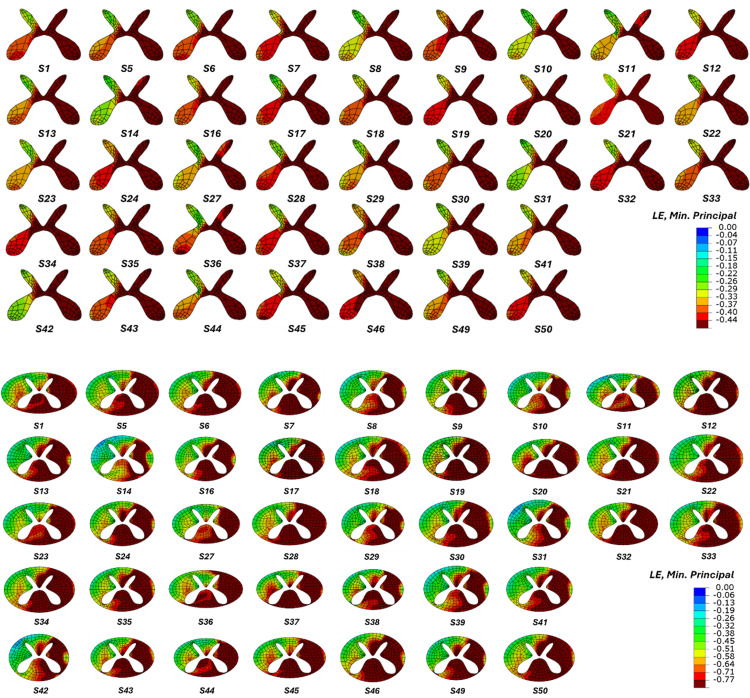
Subject-specific tissue-level biomechanics under the same loading. Applying the same impact protocol to each subject generated different tissue-level strains in each subject, as indicated by the minimum principal logarithmic strain (LE). In both the gray and white matter, the dark red area marks regions that exceed injury threshold levels and are predicted to become damaged.

In most subjects, the gray matter on the ipsilateral side (the side of the impactor) was predicted to be entirely damaged, except in Subjects 10, 11, 14, 27, and 36, where the strain levels remained close to the injury threshold (Fig 5). When assessing morphology, these subjects either had a large CSF area, i.e., greater than 35 mm^2^ (S10, S11, S14, and S27) or a relatively small cord diameter (S11, S27, and S36) in the anterior-posterior direction. In the white matter, our models predicted variability in tissue damage on the ipsilateral side. The lesion seldom extended to the lateral edge of the spinal cord on the ipsilateral side, except in Subjects 1, 5, 6, 12, 28, 32, and 36, where the lesion extended across to the edge of the ipsilateral side of the white matter. The subjects were divided into two groups, based on whether the lesion extended to the lateral edge on the ipsilateral side (such as in S6 and S28) or not (such as in S14 and S42). A paired t-test showed that this lateral extension was statistically correlated with the mediolateral occlusion of the canal by the cord (SCOW/SCW), where a tighter fit (less space available for the cord) resulted in greater extension to the edge. Increased lateral extension of the lesion was most strongly correlated with higher peak forces.

### Predicted Tissue Damage on the Contralateral Side

Unilateral contusions are intended to limit tissue damage to the ipsilateral side of the cord and damage on the contralateral side is undesirable. In terms of the gray matter, our model predicted that all subjects would experience damage on the ipsilateral side and the central canal. No damage was predicted in the contralateral dorsal horn of the gray matter in any subject (Fig 5). However, the models predicted that a few subjects would also experience tissue damage on the contralateral side of the gray matter, particularly in the ventral horn, with the most tissue damage predicted for S20, S34, and S46. These subjects all had low amounts of cerebrospinal fluid and a high occlusion of the canal in the mediolateral direction (SCOW/SCW), increasing tissue engagement. In the contralateral white matter, the dorsal columns were not predicted to be damaged, but damage in the ventral columns was often predicted in varying amounts, with the exception of Subjects S8, 10, 11, 14, 18, 22, 27, 29, 31, 39, and 42. The area of the CSF was higher in each of these subjects, indicating that a high CSF area reduced the potential for tissue damage extending to the contralateral side.

## Discussion

Pre-clinical models of SCI have recognized that intersubject differences in spinal cord and canal morphology influence both experimental and neurological outcomes [4]. Retrospective clinical studies similarly show that individuals with certain morphologies are more susceptible to severe injuries [34]. Despite this, computational models of SCI do not yet account for natural anatomical variability. In contrast, computational studies in other fields, such as knee and spine biomechanics, routinely incorporate morphological diversity to better capture intersubject differences [35,36]. In this study, we use a digital population of NHPs to investigate the role of spinal cord and canal morphology on impact and tissue-level biomechanics in a unilateral contusion model of spinal cord injury. To our knowledge, this is the first study in which such a large population of geometries has been used in a computational model of the spinal cord with the aim of making computational and experimental models of spinal cord injury more robust and comparable to experimentally observed variability in morphology [9].

The model has a few limitations. Firstly, while the impact biomechanics have been compared to the peak force values seen in experiments and validated in our prior studies [22,28], the specific tissue-damage patterns have not been validated. While this is a limitation of this study, it does not deviate from the findings of the study that morphological changes alter how external loading translates into the spinal cord tissues [13,17]. Future work should validate these findings against experiments where possible. The model also uses a simplified geometry compared to a complex subject-specific geometry, which is also common practice [13] and allows us to conduct large-scale studies such as this one with computational efficiency. The complexity of these models can be increased in future studies, where additional morphological parameters can be included.

A digital population of forty (*N = 40*) different NHP subjects was used to demonstrate that even if the same impact conditions are applied to each subject, morphological variability can result in different outcomes. This is because under the same impact loading, differences in morphology may cause differences in cord displacement and compression ratio and altering the strain patterns generated within the spinal cord, as demonstrated in prior studies [13]. However, in unilateral contusions, the influence of morphology is more nuanced than compression ratio alone, as the cord can undergo lateral slippage when only half of its geometry is impacted. In this study, differences in morphology under the same impact protocol resulted in a 15% coefficient of variation in peak forces. Our most interesting observations are the outliers in the study. Some of these subjects experienced forces as low as 12.9 N, while others experienced forces as high as 32.7 N, leading to a 20 N difference in observed peak forces attributed to morphology alone. Understanding the underlying morphology that leads to these impact force outliers is critical. A force of 12.9 N generated stress/strain patterns that are likely to produce no functional deficits in an animal based on comparisons with similar forces in our contusion experiments [5], making it difficult to assess any treatment effects. Conversely, animals with very high impact forces (30 N) are likely to have severe and contralateral injuries, that could likely result in significant adverse events such as requiring the subject to be euthanized. This is evident from our prior experimental contusions where subjects experiencing forces as high as 23 N exhibited contralateral extension of the lesion. In both cases, these outcomes would jeopardize the ethical approvals of the study and have significant adverse events. Our study highlights that preventing these forces would require that we adjust the impact parameters for these animals, or that these animals should be removed from the study.

The peak forces generated from the impact were dependent on the morphology of the subject and correlated with the amount of cerebrospinal fluid in the subject, which has been previously recognized for its role in the mechanical protection of the spinal cord [34,37]. Interestingly, our study found that the mediolateral diameter of the spinal cord and how the spinal cord fit within the spinal canal mediolaterally was more important than its anterior-posterior geometry. If a higher proportion of the spinal canal was occupied by the spinal cord, the forces generated during the impact were higher. In prior human studies, cord mismatch has also been identified as a risk factor for spinal cord injury, where a higher canal occlusion results in worse SCI [34]. The peak forces correlated with the white matter sparing, emphasizing the importance of impact biomechanics as an indicator of tissue damage, but there was no statistical correlation between peak force and gray matter sparing. Similar trends were reported by Kim et al. (2019), where peak forces during porcine contusion experiments were correlated with histologically observed white matter sparing (r = −0.714), with no statistical correlation with gray matter sparing (r = −0.429) [4]. Variability in the white matter sparing would significantly alter functional outcomes, with some subjects retaining more functions than others, and making it difficult to assess treatment effects.

Our study emphasized the important role of subject morphology on outcomes. Throughout the study, there was a recurring theme of the importance of the cerebrospinal fluid and its mechanical protection of the spinal cord, which aligns with prior studies emphasizing its role in energy dissipation [4,35]. Subjects with a higher cerebrospinal fluid area exhibited a reduction in peak forces, predicted lower white and gray matter damage, and reduced contralateral extension of the lesion. When conducting contusion experiments in subjects with increased cerebrospinal fluid, the impact parameters must be modified to combat this mechanical protection and ensure that similar results are obtained. Our results also emphasized the importance of the fit of the spinal cord within the spinal canal, particularly in the mediolateral direction. High mediolateral cord to canal occlusion ratios (SCOW/SCW) resulted in higher peak forces, predicted more damage in the ipsilateral side of the cord, with full lateral extension of the lesion in the white matter. Our models also predicted that having either a large CSF area or relatively small cord diameter in the anterior-posterior direction (SCOD) would prevent a subject from having a completely damaged gray matter on the ipsilateral side, leaving some spared tissue. This may correlate with the mechanical protection provided by the cerebrospinal fluid, or having a smaller spinal cord, which leads to poor tissue engagement during impact due to inadequate tissue and/or lateral slippage of the spinal cord away from the impact.

Subjects with this morphology also showed contralateral extension of the lesion in the gray matter. Our prior study has shown that during impact, due to its geometry, the spinal cord has a tendency of slipping laterally during a unilateral impact [16]. When there was less space available for lateral motion due to higher mediolateral canal occlusion, lateral slippage was decreased, increasing engagement of the spinal cord tissues during the impact, and resulting in higher forces. Comparatively, a smaller spinal cord relative to the spinal canal would cause the impactor to push the cord laterally, inducing lateral slippage and reduced tissue engagement, causing the cord to experience lower impact forces. We suspect that the spinal cord with a higher mediolateral diameter, the impactor better entraps the spinal cord, i.e., the entire diameter of the impactor is engaged with spinal cord tissue, with some tissue remaining on the lateral edge of the impactor. Comparatively, in a smaller spinal cord, there could be simply inadequate tissue on the lateral edge of the impactor. In these conditions, our prior studies have shown that it may be beneficial to use an impactor with a smaller diameter, allowing for better tissue engagement along the entire impactor surface [16].

The anterior-posterior morphology had little effect on impact or tissue-level biomechanics. One possible explanation for this is that our experimental protocol, which formed the base of this simulation, consisted of a preload phase, where the anterior-posterior CSF was displaced prior to the impact and the spinal cord was entrapped against the ventral surface of the spinal canal. Therefore, the effect of the anterior-posterior CSF geometry variability was virtually eliminated. In experimental protocols without this preload phase, it is possible that the anterior-posterior cerebrospinal fluid and geometry would have a more significant contribution to the outcomes.

Overall, this study demonstrates that intersubject morphological variability can have a substantial influence on impact and tissue-level biomechanics. By capturing these effects within a digital population, our approach provides a more holistic understanding of how biomechanics vary with morphology compared to a single geometry. This enables population-level questions to be addressed, offering a more realistic view of how standardized experimental conditions intersect with individual anatomy and influence outcomes.

## Conclusions

This study investigated the role of spinal cord and canal morphology on the impact and tissue-level biomechanics and predicted tissue damage in a digital population of NHPs. We found that intersubject morphological variability, which appears inherently in nature in both animals and humans, has important implications on biomechanics, with morphological variability between subjects changing the peak forces from 13 N to 33 N. Both these values would be detrimental to a NHP study, resulting in either no functional deficits that could limit our ability to detect treatment effects or result in a contralateral impact. This variability emphasizes that morphology is a key factor affecting biomechanics and that it should be included into computational studies, particularly in parametric studies, where findings may depend on the morphology selected for the analysis. Incorporating this variability into parametric computational studies is essential in making findings from computational models more significant and robust.

Our models highlighted the mechanical protection of the cord by the cerebrospinal fluid, demonstrating that subjects with a higher CSF volume exhibited reduced peak forces and more tissue sparing. This difference in tissue sparing would be seen as variability in functional outcomes, making it difficult to assess treatment efficacy. We also found that mediolateral cord-to-canal occlusion was an important predictor of outcomes, where subjects with less space available for the cord had higher peak forces and less tissue sparing. These subjects also had a higher tendency of contralateral extension of the lesion. We suspect that this may be due to poor tissue engagement and/or lateral slippage of the cord and could be improved by using a smaller impactor. Our findings emphasize that subject-specific morphological factors have significant implications. This highlights that either impact parameters need to be adjusted for morphological variability or that animals should be non-invasively pre-screened for cord/column morphology, which can be prohibitively expensive. Future work is needed to determine how to scale these impact parameters for different morphologies.

## Supporting information

S1 Table Subject-specific biomechanics and morphological parameters used in the simulation. (PDF)

S2 File Software Code. A PDF copy of the python code to be compiled in Abaqus to generate variable spinal and spinal cord morphologies.
(PDF)
