# Supplementary material for: Effect of morphology on the biomechanics of contusion models of non-human primate spinal cord injury: a finite element study in a digital population
Source: PLoS One. 2026 Feb 4;21(2):e0337794. doi: 10.1371/journal.pone.0337794 (PMC12872009; doi:10.1371/journal.pone.0337794)
Supplement: S1 Table — (PDF) [file pone.0337794.s001.pdf]

S1 Table: Supplementary Data

Subject Morphology

Subject	SCOW [mm]	SCW [mm]	SCOD [mm]	SCD [mm]	SCOW/ SCW	SCOD/ SCD	SCOD/ SCD	CSF ML	CSF AP	SCO Area	SC Area	SCO/ SC	CSF Area
S1	10.99	11.87	5.93	8.33	0.93	0.71	0.54	0.88	2.40	51.18	77.66	0.66	26.47
S5	10.38	12.33	6.47	8.19	0.84	0.79	0.62	1.95	1.72	52.75	79.31	0.67	26.57
S6	10.54	11.63	6.49	8.37	0.91	0.78	0.62	1.09	1.88	53.72	76.45	0.70	22.73
S7	8.74	10.14	6.19	7.66	0.86	0.81	0.71	1.40	1.47	42.49	61.00	0.70	18.51
S8	9.57	12.53	6.43	8.26	0.76	0.78	0.67	2.96	1.83	48.33	81.29	0.59	32.96
S9	8.94	10.74	6.68	8.46	0.83	0.79	0.75	1.80	1.78	46.90	71.36	0.66	24.46
S10	9.09	12.4	6.05	8.06	0.73	0.75	0.67	3.31	2.01	43.19	78.50	0.55	35.30
S11	10.59	12.85	5.5	8.81	0.82	0.62	0.52	2.26	3.31	45.75	88.91	0.51	43.17
S12	9.13	11.14	6.24	7.8	0.82	0.80	0.68	2.01	1.56	44.75	68.24	0.66	23.50
S13	9.34	12.32	6.55	7.53	0.76	0.87	0.70	2.98	0.98	48.05	72.86	0.66	24.81
S14	9.03	12.86	6.65	8.17	0.70	0.81	0.74	3.83	1.52	47.16	82.52	0.57	35.36
S16	9.03	11.22	6.61	8.07	0.80	0.82	0.73	2.19	1.46	46.88	71.11	0.66	24.24
S17	9.53	10.12	6.03	8.39	0.94	0.72	0.63	0.59	2.36	45.13	66.69	0.68	21.55
S18	10.99	12.31	6.52	8.89	0.89	0.73	0.59	1.32	2.37	56.28	85.95	0.65	29.67
S19	10	11.09	6.55	8.19	0.90	0.80	0.66	1.09	1.64	51.44	71.34	0.72	19.89
S20	9.72	10.28	5.68	8.33	0.95	0.68	0.58	0.56	2.65	43.36	67.26	0.64	23.89
S21	10.68	11.48	6.58	7.89	0.93	0.83	0.62	0.80	1.31	55.19	71.14	0.78	15.95
S22	10.52	12.97	6.82	8.06	0.81	0.85	0.65	2.45	1.24	56.35	82.10	0.69	25.75
S23	10.38	12.74	6.11	7.82	0.81	0.78	0.59	2.36	1.71	49.81	78.25	0.64	28.44
S24	9.24	11.04	6.39	8.29	0.84	0.77	0.69	1.80	1.90	46.37	71.88	0.65	25.51
S27	9.78	12.84	5.67	8.06	0.76	0.70	0.58	3.06	2.39	43.55	81.28	0.54	37.73
S28	10.97	11.15	6.35	7.68	0.98	0.83	0.58	0.18	1.33	54.71	67.26	0.81	12.54
S29	8.94	11.99	6.16	7.91	0.75	0.78	0.69	3.05	1.75	43.25	74.49	0.58	31.24
S30	10.86	11.83	6.82	8.99	0.92	0.76	0.63	0.97	2.17	58.17	83.53	0.70	25.36
S31	9.01	12.89	6.71	8.16	0.70	0.82	0.74	3.88	1.45	47.48	82.61	0.57	35.13
S32	10.28	10.7	6.28	7.52	0.96	0.84	0.61	0.42	1.24	50.70	63.20	0.80	12.49

S33	10.7	12.75	6.8	8.87	0.84	0.77	0.64	2.05	2.07	57.15	88.82	0.64	31.68
S34	10.5	11.03	6.78	8.48	0.95	0.80	0.65	0.53	1.70	55.91	73.46	0.76	17.55
S35	9.93	11.69	6.74	8.84	0.85	0.76	0.68	1.76	2.10	52.57	81.16	0.65	28.60
S36	10.96	12.93	5.57	7.55	0.85	0.74	0.51	1.97	1.98	47.95	76.67	0.63	28.73
S37	9.78	10.13	5.61	7.79	0.97	0.72	0.57	0.35	2.18	43.09	61.98	0.70	18.89
S38	8.83	10.69	5.9	8.57	0.83	0.69	0.67	1.86	2.67	40.92	71.95	0.57	31.04
S39	9.34	12.42	6.6	8.47	0.75	0.78	0.71	3.08	1.87	48.42	82.62	0.59	34.21
S41	9.99	12.38	6.77	7.81	0.81	0.87	0.68	2.39	1.04	53.12	75.94	0.70	22.82
S42	8.69	12.66	6.83	8.28	0.69	0.82	0.79	3.97	1.45	46.62	82.33	0.57	35.71
S43	9.43	10.63	5.95	8.23	0.89	0.72	0.63	1.20	2.28	44.07	68.71	0.64	24.64
S44	9.53	11.74	5.74	7.85	0.81	0.73	0.60	2.21	2.11	42.96	72.38	0.59	29.42
S45	10.52	11.35	5.92	8.44	0.93	0.70	0.56	0.83	2.52	48.91	75.24	0.65	26.32
S46	8.83	10.2	6.69	7.98	0.87	0.84	0.76	1.37	1.29	46.40	63.93	0.73	17.53
S49	8.5	11.24	6.28	7.73	0.76	0.81	0.74	2.74	1.45	41.92	68.24	0.61	26.31
S50	10.19	11.02	6.78	8.13	0.92	0.83	0.67	0.83	1.35	54.26	70.37	0.77	16.10

Summary of Biomechanics

Subject	Peak Force	Impulse	Spared GM	Spared WM
S1	23.20	0.45	43%	54%
S5	28.22	0.54	44%	55%
S6	26.32	0.54	43%	50%
S7	22.38	0.46	36%	50%
S8	23.68	0.42	47%	66%
S9	22.27	0.45	35%	53%
S10	21.59	0.42	48%	71%
S11	19.48	0.38	54%	66%
S12	27.71	0.54	34%	45%
S13	25.51	0.52	43%	54%
S14	12.89	0.27	49%	79%
S16	23.83	0.48	42%	53%
S17	24.12	0.47	38%	68%
S18	23.12	0.45	43%	60%
S19	25.97	0.51	36%	47%
S20	22.01	0.43	24%	40%
S21	29.61	0.59	39%	48%
S22	26.53	0.53	43%	55%
S23	26.09	0.51	47%	61%
S24	25.04	0.49	38%	52%
S27	22.53	0.44	53%	69%
S28	32.68	0.64	54%	46%
S29	22.03	0.44	46%	63%
S30	24.31	0.50	44%	53%
S31	19.78	0.40	44%	67%
S32	31.58	0.63	34%	44%
S33	25.40	0.51	43%	58%
S34	28.30	0.56	35%	46%
S35	24.82	0.49	42%	53%
S36	29.33	0.56	46%	52%
S37	27.18	0.51	43%	46%
S38	18.38	0.37	43%	58%
S39	20.89	0.41	43%	67%
S41	24.47	0.49	43%	57%
S42	18.28	0.37	43%	65%
S43	22.09	0.42	43%	53%
S44	22.17	0.44	48%	58%
S45	23.78	0.46	41%	53%
S46	24.32	0.46	25%	48%
S49	25.83	0.49	42%	54%
S50	29.18	0.56	35%	48%
