# Supplementary material for: Effect of morphology on the biomechanics of contusion models of non-human primate spinal cord injury: a finite element study in a digital population
Source: PLoS One. 2026 Feb 4;21(2):e0337794. doi: 10.1371/journal.pone.0337794 (PMC12872009; doi:10.1371/journal.pone.0337794)
Supplement: S2 File — A PDF copy of the python code to be compiled in Abaqus to generate variable spinal and spinal cord morphologies. (PDF) [file pone.0337794.s002.pdf]

```
#SI units are consistently used throughout the model

from part import *
from material import *
from section import *
from assembly import *
from step import *
from interaction import *
from load import *
from mesh import *
from optimization import *
from job import *
from sketch import *
from visualization import *
from connectorBehavior import *
from datum import*
import random


#---------------------------------------------------------PART CREATION---------------------------------------------------------

#All variables are written here

PiaThickness=1E-6*150 #The pia thickness is 350 microns
DuraThickness=1E-6*350 #The dura thickness is 350 microns
SCOD=1E-3*1E-2*random.randrange(550,700,1) #The AP diameter of the cord is between 5.5-7 mm
SCOW=1E-3*1E-2*random.randrange(850,1100,1) #The ML diameter of the cord is between 8.5-11 mm
SCD=1E-3*1E-2*random.randrange(750,900,1) #The AP diameter of the canal is between 7.5-9 mm
SCW=1E-3*1E-2*random.randrange(1000,1300,1) #The ML diameter of the canal is between 10-13 mm
LengthC4=1E-3*1E-2*880 #This defines the extruded length of C4
LengthC5=1E-3*1E-2*852 #This defines the extruded length of C5
LengthC6=1E-3*1E-2*828 #This defines the extruded length of C6
LengthIVDH45=1E-3*1E-2*118 #This defines the space between C4 and C5
LengthIVDH56=1E-3*1E-2*120 #This defines the space between C5 and C6
CordLength=LengthC4+LengthC5+LengthC6+LengthIVDH45+LengthIVDH56 #The total cord length is a summation of the VB heights and their spacing
MLAlignment=0.5E-3 #The mediolateral aligmment of the impactor is set to 0.5 mm
ImpactorSize=5E-3 #The diameter of the impactor is set to 5 mm
offset=DuraThickness*1.2  #This offset is defined to ensure that there are no contact overclosures attributed to meshing near adjacent geometries

### SPINAL CORD: Creates an ellipse with axisPoint 1 corresponding to the radius on the horizontal axis (SCOW), and axisPoint2 represents the radius on the vertical axis (SCOD). Extrude depth corresponds to the length of the segment being modelled

mdb.models['Model-1'].ConstrainedSketch(name='__profile__', sheetSize=0.01)
mdb.models['Model-1'].sketches['__profile__'].sketchOptions.setValues(
    decimalPlaces=5)
mdb.models['Model-1'].sketches['__profile__'].EllipseByCenterPerimeter(center=(0.0, 0.0),
    axisPoint1=(SCOW/2, 0.0),axisPoint2=(0.0,SCOD/2))
mdb.models['Model-1'].Part(dimensionality=THREE_D, name='Cord', type=
    DEFORMABLE_BODY)
mdb.models['Model-1'].parts['Cord'].BaseSolidExtrude(depth=CordLength, sketch=
    mdb.models['Model-1'].sketches['__profile__'])
del mdb.models['Model-1'].sketches['__profile__']

### Creates the spinal canal

### C4
mdb.models['Model-1'].ConstrainedSketch(name='__profile__', sheetSize=0.02)
mdb.models['Model-1'].sketches['__profile__'].sketchOptions.setValues(decimalPlaces=4)
```

```
mdb.models['Model-1'].sketches['__profile__'].EllipseByCenterPerimeter(center=(0.0, 0.0),axisPoint1=(SCW/2+offset, 0.0),axisPoint2=(0.0,offset+SCD/2))
mdb.models['Model-1'].Part(dimensionality=THREE_D, name='C4', type=DISCRETE_RIGID_SURFACE)
mdb.models['Model-1'].parts['C4'].BaseShellExtrude(depth=LengthC4, sketch=mdb.models['Model-1'].sketches['__profile__'])
del mdb.models['Model-1'].sketches['__profile__']

### C5
mdb.models['Model-1'].ConstrainedSketch(name='__profile__', sheetSize=0.02)
mdb.models['Model-1'].sketches['__profile__'].sketchOptions.setValues(decimalPlaces=4)
mdb.models['Model-1'].sketches['__profile__'].EllipseByCenterPerimeter(center=(0.0, 0.0),axisPoint1=(SCW/2+offset*1.1, 0.0),axisPoint2=(0.0,offset*1.1+SCD/2))
mdb.models['Model-1'].sketches['__profile__'].Line(point1=(-0.0025,10E-3), point2=(-0.0025, 0.0))
mdb.models['Model-1'].sketches['__profile__'].Line(point1=(0.0, 0.002), point2=(10E-3, 0.002))
mdb.models['Model-1'].sketches['__profile__'].autoTrimCurve(curve1=
    mdb.models['Model-1'].sketches['__profile__'].geometry[2], point1=(
    (((SCW/2+offset*1.1)**2)*(1-(0.004**2)/((SCD/2+offset*1.1)**2)))**0.5, 0.004))
mdb.models['Model-1'].sketches['__profile__'].delete(objectList=(
    mdb.models['Model-1'].sketches['__profile__'].geometry[4],
    mdb.models['Model-1'].sketches['__profile__'].geometry[5]))
mdb.models['Model-1'].Part(dimensionality=THREE_D, name='C5', type=DISCRETE_RIGID_SURFACE)
mdb.models['Model-1'].parts['C5'].BaseShellExtrude(depth=LengthC5, sketch=mdb.models['Model-1'].sketches['__profile__'])
del mdb.models['Model-1'].sketches['__profile__']

### C6
mdb.models['Model-1'].ConstrainedSketch(name='__profile__', sheetSize=0.02)
mdb.models['Model-1'].sketches['__profile__'].sketchOptions.setValues(decimalPlaces=4)
mdb.models['Model-1'].sketches['__profile__'].EllipseByCenterPerimeter(center=(0.0, 0.0),axisPoint1=(SCW/2+offset, 0.0),axisPoint2=(0.0,offset+SCD/2))
mdb.models['Model-1'].Part(dimensionality=THREE_D, name='C6', type=DISCRETE_RIGID_SURFACE)
mdb.models['Model-1'].parts['C6'].BaseShellExtrude(depth=LengthC6, sketch=mdb.models['Model-1'].sketches['__profile__'])
del mdb.models['Model-1'].sketches['__profile__']

mdb.models['Model-1'].parts['C4'].setValues(space=THREE_D, type=
    DISCRETE_RIGID_SURFACE)
mdb.models['Model-1'].parts['C5'].setValues(space=THREE_D, type=
    DISCRETE_RIGID_SURFACE)
mdb.models['Model-1'].parts['C6'].setValues(space=THREE_D, type=
    DISCRETE_RIGID_SURFACE)

### CSF: Create CSF as the difference between the SCD-SCOD and SCW-SCOW.
mdb.models['Model-1'].ConstrainedSketch(name='__profile__', sheetSize=0.1)
mdb.models['Model-1'].sketches['__profile__'].sketchOptions.setValues(
    decimalPlaces=3)
mdb.models['Model-1'].sketches['__profile__'].EllipseByCenterPerimeter(center=(0.0, 0.0),
    axisPoint1=(SCW/2, 0.0),axisPoint2=(0.0,SCD/2))
mdb.models['Model-1'].sketches['__profile__'].EllipseByCenterPerimeter(center=(0.0, 0.0),
    axisPoint1=(SCOW/2, 0.0),axisPoint2=(0.0,SCOD/2))
mdb.models['Model-1'].Part(dimensionality=THREE_D, name='CSF', type=DEFORMABLE_BODY)
mdb.models['Model-1'].parts['CSF'].BaseSolidExtrude(depth=CordLength, sketch=
    mdb.models['Model-1'].sketches['__profile__'])
del mdb.models['Model-1'].sketches['__profile__']

### DURA: It will be a shell starting from SCOD and SCOW, and then the DuraShell thickness.
mdb.models['Model-1'].ConstrainedSketch(name='__profile__', sheetSize=0.2)
mdb.models['Model-1'].sketches['__profile__'].sketchOptions.setValues(
    decimalPlaces=3)
```

```
mdb.models['Model-1'].sketches['__profile__'].EllipseByCenterPerimeter(center=(0.0, 0.0),
    axisPoint1=(SCW/2, 0.0),axisPoint2=(0.0,SCD/2))
mdb.models['Model-1'].Part(dimensionality=THREE_D, name='Dura', type=
    DEFORMABLE_BODY)
mdb.models['Model-1'].parts['Dura'].BaseShellExtrude(depth=CordLength, sketch=
    mdb.models['Model-1'].sketches['__profile__'])
del mdb.models['Model-1'].sketches['__profile__']
mdb.models['Model-1'].parts['Dura'].Surface(name='DuraIn', side2Faces=
    mdb.models['Model-1'].parts['Dura'].faces.getSequenceFromMask(('[#1 ]', ),
    ))
mdb.models['Model-1'].parts['Dura'].Surface(name='DuraOut', side1Faces=
    mdb.models['Model-1'].parts['Dura'].faces.getSequenceFromMask(('[#1 ]', ),
    ))
mdb.models['Model-1'].parts['Dura'].Surface(name='DuraAll', side12Faces=
    mdb.models['Model-1'].parts['Dura'].faces.getSequenceFromMask(('[#1 ]', ),
    ))

##CREATE PIA AS AN INWARD SKIN ON THE CORD
mdb.models['Model-1'].parts['Cord'].Skin(faces=
    mdb.models['Model-1'].parts['Cord'].faces.getSequenceFromMask(('[#1 ]', ),
    ), name='Pia')

#CREATE IMPACTOR
mdb.models['Model-1'].ConstrainedSketch(name='__profile__', sheetSize=0.001)
mdb.models['Model-1'].sketches['__profile__'].sketchOptions.setValues(
    decimalPlaces=5)
mdb.models['Model-1'].sketches['__profile__'].ConstructionLine(point1=(0.0,
    -0.0005), point2=(0.0, 0.0005))
mdb.models['Model-1'].sketches['__profile__'].FixedConstraint(entity=
    mdb.models['Model-1'].sketches['__profile__'].geometry[2])
mdb.models['Model-1'].sketches['__profile__'].rectangle(point1=(0.0, 0.006),
    point2=(ImpactorSize/2, 0.0))
mdb.models['Model-1'].sketches['__profile__'].FilletByRadius(curve1=
    mdb.models['Model-1'].sketches['__profile__'].geometry[5], curve2=
    mdb.models['Model-1'].sketches['__profile__'].geometry[4], nearPoint1=(
    0.00200544763356447, 0.000265653477981687), nearPoint2=(
    0.00167165545281023, -1.95987522602081e-05), radius=0.0001)
mdb.models['Model-1'].Part(dimensionality=THREE_D, name='Impactor', type=
     DISCRETE_RIGID_SURFACE)
mdb.models['Model-1'].parts['Impactor'].BaseSolidRevolve(angle=360.0,
    flipRevolveDirection=OFF, sketch=
    mdb.models['Model-1'].sketches['__profile__'])
del mdb.models['Model-1'].sketches['__profile__']

#Move the sketch to the correct position
mdb.models['Model-1'].ConstrainedSketch(name='__edit__', objectToCopy=
    mdb.models['Model-1'].parts['Impactor'].features['Solid revolve-1'].sketch)
mdb.models['Model-1'].parts['Impactor'].projectReferencesOntoSketch(filter=
    COPLANAR_EDGES, sketch=mdb.models['Model-1'].sketches['__edit__'],
    upToFeature=
    mdb.models['Model-1'].parts['Impactor'].features['Solid revolve-1'])
mdb.models['Model-1'].sketches['__edit__'].move(objectList=(
    mdb.models['Model-1'].sketches['__edit__'].geometry[2],
    mdb.models['Model-1'].sketches['__edit__'].geometry[3],
    mdb.models['Model-1'].sketches['__edit__'].geometry[4],
    mdb.models['Model-1'].sketches['__edit__'].geometry[5],
    mdb.models['Model-1'].sketches['__edit__'].geometry[6],
    mdb.models['Model-1'].sketches['__edit__'].geometry[7]), vector=(0.0, SCD/2+offset))
mdb.models['Model-1'].parts['Impactor'].features['Solid revolve-1'].setValues(
    sketch=mdb.models['Model-1'].sketches['__edit__'])
del mdb.models['Model-1'].sketches['__edit__']
mdb.models['Model-1'].parts['Impactor'].regenerate()

##Convert solid rigid body of the impactor to a shell
mdb.models['Model-1'].parts['Impactor'].RemoveCells(cellList=
    mdb.models['Model-1'].parts['Impactor'].cells.getSequenceFromMask(mask=(
```

```
    '[#1 ]', ), ))

##SECTIONS CREATED FOR PIA AS A SKIN
mdb.models['Model-1'].HomogeneousShellSection(idealization=NO_IDEALIZATION,
    integrationRule=SIMPSON, material='Pia', name='Section-Pia', nodalThicknessField=
    '', numIntPts=5, poissonDefinition=DEFAULT, preIntegrate=OFF, temperature=
    GRADIENT, thickness=PiaThickness, thicknessField='', thicknessModulus=None,
    thicknessType=UNIFORM, useDensity=OFF)
mdb.models['Model-1'].parts['Cord'].Set(cells=
    mdb.models['Model-1'].parts['Cord'].cells.getSequenceFromMask(('[#1 ]', ),
    ), name='Set-Cord')
mdb.models['Model-1'].parts['Cord'].Set(name='Set-Pia', skinFaces=(('Pia',
    mdb.models['Model-1'].parts['Cord'].faces.getSequenceFromMask(('[#1 ]', ),
    )), ))
mdb.models['Model-1'].parts['Cord'].SectionAssignment(offset=0.0, offsetField=
    '', offsetType=BOTTOM_SURFACE, region=
    mdb.models['Model-1'].parts['Cord'].sets['Set-Pia'], sectionName=
    'Section-Pia', thicknessAssignment=FROM_SECTION)

##ASSEMBLY STARTS - INSTANCE CREATIONS
mdb.models['Model-1'].rootAssembly.DatumCsysByDefault(CARTESIAN)
mdb.models['Model-1'].rootAssembly.Instance(dependent=ON, name='C4-1', part=
    mdb.models['Model-1'].parts['C4'])
mdb.models['Model-1'].rootAssembly.Instance(dependent=ON, name='C5-1', part=
    mdb.models['Model-1'].parts['C5'])
mdb.models['Model-1'].rootAssembly.Instance(dependent=ON, name='C6-1', part=
    mdb.models['Model-1'].parts['C6'])
mdb.models['Model-1'].rootAssembly.Instance(dependent=ON, name='CSF-1', part=
    mdb.models['Model-1'].parts['CSF'])
mdb.models['Model-1'].rootAssembly.Instance(dependent=ON, name='Cord-1', part=
    mdb.models['Model-1'].parts['Cord'])
mdb.models['Model-1'].rootAssembly.Instance(dependent=ON, name='Impactor-1',
    part=mdb.models['Model-1'].parts['Impactor'])
mdb.models['Model-1'].rootAssembly.Instance(dependent=ON, name='Dura-1', part=
    mdb.models['Model-1'].parts['Dura'])

##TRANSLATE THE VB INSTANCES
mdb.models['Model-1'].rootAssembly.translate(instanceList=('C4-1', ), vector=(
    0.0, 0.0, LengthC5+LengthIVDH56+LengthIVDH45+LengthC6))
mdb.models['Model-1'].rootAssembly.translate(instanceList=('C5-1', ), vector=(
    0.0, 0.0, LengthC6+LengthIVDH56))
mdb.models['Model-1'].rootAssembly.translate(instanceList=('Impactor-1', ), vector=(
    ImpactorSize/2-MLAlignment, 0.0,0.0))
mdb.models['Model-1'].rootAssembly.translate(instanceList=('Impactor-1', ), vector=(
    0.0, 0.0, LengthC6+LengthIVDH56+ImpactorSize))

##ASSEMBLY ENDS

# OPTIONAL - CORD PARTITIONING INTO GRAY AND WHITE MATTER

##Cord Mesh
mdb.models['Model-1'].parts['Cord'].seedPart(deviationFactor=0.1,
    minSizeFactor=0.1, size=0.0002)
mdb.models['Model-1'].parts['Cord'].generateMesh()
mdb.models['Model-1'].parts['Cord'].setElementType(elemTypes=(ElemType(
    elemCode=C3D8R, elemLibrary=EXPLICIT, secondOrderAccuracy=ON,
    kinematicSplit=AVERAGE_STRAIN, hourglassControl=ENHANCED,
    distortionControl=ON, lengthRatio=0.100000001490116), ElemType(
    elemCode=C3D6, elemLibrary=EXPLICIT), ElemType(elemCode=C3D4,
    elemLibrary=EXPLICIT)), regions=(
    mdb.models['Model-1'].parts['Cord'].cells.getSequenceFromMask(('[#1 ]', ),
    ), ))

##Creating face partition of the geometry of the GM
mdb.models['Model-1'].parts['Cord'].deleteMesh(regions=
    mdb.models['Model-1'].parts['Cord'].cells.getSequenceFromMask(('[#1 ]', ),
    ))
```

```
mdb.models['Model-1'].ConstrainedSketch(gridSpacing=0.001, name='__profile__',
    sheetSize=0.063, transform=
    mdb.models['Model-1'].parts['Cord'].MakeSketchTransform(
    sketchPlane=mdb.models['Model-1'].parts['Cord'].faces[1],
    sketchPlaneSide=SIDE1,
    sketchUpEdge=mdb.models['Model-1'].parts['Cord'].edges[1],
    sketchOrientation=RIGHT, origin=(0.0, 0.0, CordLength)))
mdb.models['Model-1'].sketches['__profile__'].sketchOptions.setValues(
    decimalPlaces=5)
mdb.models['Model-1'].parts['Cord'].projectReferencesOntoSketch(filter=
    COPLANAR_EDGES, sketch=mdb.models['Model-1'].sketches['__profile__'])
mdb.models['Model-1'].sketches['__profile__'].Spline(points=((
    -0.00262715062126517, 0.00211353576742113), (-0.003, 0.002), (-0.003,
    0.001), (-0.001, -0.0005), (-0.002, -0.002), (-0.002, -0.00225), (-0.00175,
    -0.00225), (0.0, -0.000377459218725562), (0.00175, -0.00225), (0.002,
    -0.00225), (0.002, -0.002), (0.001, -0.0005), (0.003, 0.001), (
    0.00300185115395764, 0.002), (0.00257512787356973, 0.00211353576742113), (
    0.00202749207859469, 0.002), (0.000676178233232349, 0.000111017376184464),
    (-0.000647643581032753, 0.000111017376184464), (-0.002, 0.002), (
    -0.00262715062126517, 0.00211353576742113)))
mdb.models['Model-1'].sketches['__profile__'].rotate(angle=180.0, centerPoint=(
    0.0, -0.00025), objectList=(
    mdb.models['Model-1'].sketches['__profile__'].geometry[3], ))
mdb.models['Model-1'].parts['Cord'].PartitionFaceBySketch(faces=
    mdb.models['Model-1'].parts['Cord'].faces.getSequenceFromMask(('[#2 ]', ),
    ), sketch=mdb.models['Model-1'].sketches['__profile__'], sketchUpEdge=
    mdb.models['Model-1'].parts['Cord'].edges[1])
del mdb.models['Model-1'].sketches['__profile__']
mdb.models['Model-1'].ConstrainedSketch(name='__edit__', objectToCopy=
    mdb.models['Model-1'].parts['Cord'].features['Partition face-1'].sketch)
mdb.models['Model-1'].parts['Cord'].projectReferencesOntoSketch(filter=
    COPLANAR_EDGES, sketch=mdb.models['Model-1'].sketches['__edit__'],
    upToFeature=
    mdb.models['Model-1'].parts['Cord'].features['Partition face-1'])
mdb.models['Model-1'].sketches['__edit__'].move(objectList=(
    mdb.models['Model-1'].sketches['__edit__'].geometry[3], ), vector=(0.0,
    0.000372540781274438))
mdb.models['Model-1'].parts['Cord'].features['Partition face-1'].setValues(
    sketch=mdb.models['Model-1'].sketches['__edit__'])
del mdb.models['Model-1'].sketches['__edit__']
mdb.models['Model-1'].parts['Cord'].regenerate()

##Projecting the gray matter sketch across the cord length
mdb.models['Model-1'].parts['Cord'].DatumAxisByPrincipalAxis(principalAxis=
    ZAXIS)
mdb.models['Model-1'].parts['Cord'].PartitionCellByExtrudeEdge(cells=
    mdb.models['Model-1'].parts['Cord'].cells.getSequenceFromMask(('[#1 ]', ),
    ), edges=(mdb.models['Model-1'].parts['Cord'].edges[1], ), line=
    mdb.models['Model-1'].parts['Cord'].datums[8], sense=REVERSE)

#END
```
